# Marine Invertebrate Natural Products for Anti-Inflammatory and Chronic Diseases

**DOI:** 10.1155/2013/572859

**Published:** 2013-12-31

**Authors:** Kalimuthu Senthilkumar, Se-Kwon Kim

**Affiliations:** ^1^Marine Biochemistry Laboratory, Department of Chemistry and Marine Bioprocess Research Center, Pukyong National University, Busan 608-737, Republic of Korea; ^2^Department of Chemistry, Pukyong National University, Daeyeon Campus, 599-1, Nam gu, Busan, 608-737, Republic of Korea

## Abstract

The marine environment represents a relatively available source of functional ingredients that can be applied to various aspects of food processing, storage, and fortification. Moreover, numerous marine invertebrates based compounds have biological activities and also interfere with the pathogenesis of diseases. Isolated compounds from marine invertebrates have been shown to pharmacological activities and are helpful for the invention and discovery of bioactive compounds, primarily for deadly diseases like cancer, acquired immunodeficiency syndrome (AIDS), osteoporosis, and so forth. Extensive research within the last decade has revealed that most chronic illnesses such as cancer, neurological diseases, diabetes, and autoimmune diseases exhibit dysregulation of multiple cell signaling pathways that have been linked to inflammation. On the basis of their bioactive properties, this review focuses on the potential use of marine invertebrate derived compounds on anti-inflammatory and some chronic diseases such as cardiovascular disease, osteoporosis, diabetes, HIV, and cancer.

## 1. Introduction

Chronic diseases constitute a major cause of mortality and the World Health Organization (WHO) reports chronic noncommunicable conditions to be the leading cause of mortality in the world, representing 35 million deaths in 2005 and over 60% of all deaths. Each year, more than 30 million deaths (52% of all deaths) by chronic diseases include cardiovascular disease (30%), cancer (13%), chronic respiratory disease (7%), and diabetes (2%). The global burden of disease resulting from all noncommunicable conditions, which includes premature death and disability, is 49%; 80% of these deaths occur in low and middle income countries [1, 2]. Increasing knowledge regarding the impact of diet on human health along with the state-of-the-art technologies has led to significant nutritional discoveries, product innovations, and mass production on unprecedented scale [3]. In particular, naturally occurring bioactive extracts or single compounds are believed to benefit human health and research resulting in substantial advances in nutritional knowledge. There is also growing awareness that dietary source and form of food may affect overall health. Suitably, the role of food as an agent for improving health has been recognized, initiating the development of new classes of compound from marine environment [4].

The marine world, due to its phenomenal biodiversity, is a rich natural resource of many biologically active compounds such as polyunsaturated fatty acids (PUFAs), sterols, proteins, polysaccharides, antioxidants, and pigments. Many marine organisms live in complex habitats exposed to extreme conditions and, in adapting to new environmental surroundings, they produce a wide variety of secondary (biologically active) metabolites which cannot be found in other organisms. Moreover, considering its great taxonomic diversity, investigation related to the search of new bioactive compounds from the marine environment has seen in almost unlimited field [5, 6]. Marine-based bioactive food ingredients can be derived from many sources, including marine plants, microorganisms, and sponges, all of which contain their own unique set of biomolecules [5]. However, these naturally occurring bioactive substances has defined health benefit on the human body significantly [3]. Therefore, this review discusses the existing scientific knowledge which demonstrates the suitability of marine derived bioactive compounds for the prevention and treatment of anti-inflammatory and chronic diseases.

## 2. Marine Environment and Availability of Natural Products

**Figure 1:**
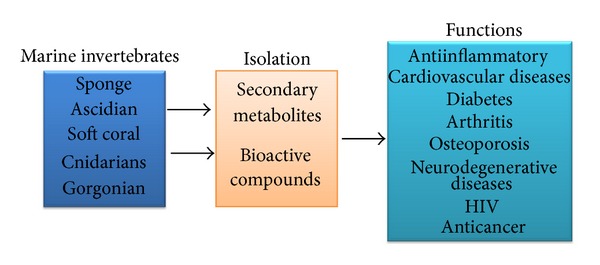
Summary of marine invertebrate natural products with anti-inflammatory and some chronic diseases.

Marine biotechnology is the science in which marine organisms are used in full or partially to make or modify products for specific uses. The different molecular and biotechnological methods elucidated for isolating natural products from aquatic and terrestrial organisms. A remarkable number of new natural products (NPs) have been isolated from various marine sources in the past decades [7]. In recent years, many bioactive compounds have been extracted from marine invertebrates such as sponges, tunicates, bryozoans, and mollusca, [8, 9]. The marine environment covers a wide thermal range including pressure range (1–1000 atm) and nutrient range (oligotrophic to eutrophic) and has extensive photic and nonphotic zones [10]. But with the development of new diving techniques, remote operated machines, and so forth, it is possible to collect marine organisms and during the past decade, over 5000 novel compounds have been isolated from shallow waters to 900-m depths of the sea [11]. The knowledge of the physiological and biochemical features of marine organisms might contribute to the identification of natural products of biomedical importance. Natural products have been the source of most of the active ingredients of medicines. This is widely accepted to be true when applied to drug discovery in “olden times” before the advent of high-throughput screening and the postgenomic era: more than 80% of drug substances were natural products or inspired by a natural compound [12]. However, comparisons of the information presented on sources of new drugs from 1981 to 2007 and 2010 [13–15] indicate that almost half of the drugs approved since 1994 are based on natural products including marine invertebrates. Marine invertebrates synthesize primary and secondary metabolites that are ultimately screened and described by researchers as NPs. Primary metabolites include amino acids, simple sugars, nucleic acids, and lipids. Secondary metabolites, such as alkaloids, terpenoids, and other compounds, have known bioactivities and biological functions [16]. Thus, marine invertebrate derived compounds may be useful as an alternative medicine for various diseases.  Figure 1 provides an overview of this review.

## 3. Anti-inflammatory Activity

Inflammation is part of the complex biological response of vascular tissues to harmful stimuli, such as pathogens, damaged cells, or irritants. The signs of acute inflammation are pain, heat, redness, swelling, and loss of function. Inflammations has different names in different parts of the body asthma (inflammation of the airways), arthritis (inflammation of the joints), dermatitis (inflammation of the skin), and so on. Inflammation is the crucial first step in fighting infection and healing wounds. However, persistent inflammation on immune system is always activated, the condition known as chronic inflammation which leads to chronic diseases [17]. However, if the response is exaggerated, misdirected, or long term, the inflammation can adversely affect health and give rise to many conditions such as inflammatory bowel disease, arthritis, and asthma [18, 19]. Anti-inflammatory refers to the property of a substance or treatment that reduces inflammation. Many steroids and nonsteroidal anti-inflammatory drugs (NSAIDs) are widely used for the treatment for inflammation. Alternately herbal medicines also play a role in inflammation apart from that marine derived bioactive compounds showed anti-inflammatory actions. Marine invertebrates are one of the major groups of biological organisms (Porifera, Cnidaria, Mollusca, Arthropoda, Echinodermata, and so forth) that gave until now significant number of natural products and secondary metabolites with pharmacological properties and lead in the formulation of novel drugs. These natural products have a wide range of therapeutic properties, including antimicrobial, antioxidant, antihypertensive, anticoagulant, anticancer, anti-inflammatory, wound healing and immune modulator, and other medicinal effects [20].

Alkaloid is a group of biological amine and cyclic compounds having nitrogen in the ring, naturally occurring in plant, microbes, animals, and marine organisms. Both halogenated and nonhalogenated forms have attracted researchers' interest because of their pharmaceutical importance as bioactive compounds and as biological probes for physiological studies [21]. Indole alkaloids from marine invertebrates have reported to be anti-inflammatory potentials; these include conicamin from tunicate [22], Lepadiformines A and B from ascidian [23] and aplysinopsin-type compound from sponge *Hyrtios erecta* [24], manzamine from sponge [25], carteramine A from sponge [26], and ascidiathiazones A and B [27, 28] from ascidan. The tricyclic alkaloids ascidiathiazone is isolated from *Ascidian aplidium* species that affected superoxide production by human neutrophils *in vitro *(IC_50_ = 0.44–1.55 *μ*M), as well as *ex vivo* and studies suggested that these two compounds might become “potential anti-inflammatory pharmaceutical” leads. Cyclooxygenase (COX) enzyme synthesizes prostaglandins, creating inflammation. The new cembranoids, crassumolides A and C from the soft coral *Lobophytum crassum* inhibited the expression of iNOS and COX-2 (IC_50_ less than 10 *μ*M) [29]. Also, another cembranolides durumolides A–C from the soft coral *Lobophytum duru* inhibited both iNOS and COX-2 proteins in LPS-activated RAW 264.7 cells *in vitro*, suggested that the *α*-methylene-*α*-lactone moiety of these compounds was necessary for the activity [30].

A new briarane-type diterpenoids frajunolides B and C, isolated from the Taiwanese gorgonian *Junceella fragilis*, significantly inhibited superoxide anion and elastase generation from human neutrophils *in vitro* (apparent IC_50_ greater than 10 *μ*g/mL) [31]. Manzamine A, (-)-8-hydroxymanzamine A, and hexahydro-8-hydroxymanzamine, potently inhibited thromboxane (TXB2) generation (IC_50_ = 0.25, less than 0.1, and 1.97 *μ*M, resp.) in brain microglia [25]. Kossuga et al. [32] demonstrated that the polyketide plakortide P isolated from the Brazilian sponge *P. angulospiculatus*, potently inhibited thromboxane B2 release (IC_50_ = 0.93 *μ*M) from activated rat brain microglia, appears to be a potentially “novel anti-neuroinflammatory agent”. A halogenated furanone rubrolide O isolated from a New Zealand ascidian *Synoicum sp.*, which inhibited superoxide anion production in human neutrophils (IC_50_ = 35 *μ*M)  *in vitro* with low toxicity [28]. A novel dimeric oroidin (type of alkaloid) derivative carteramine A in the marine sponge *Stylissa carteri*, showed that inhibited neutrophil chemotaxis (IC_50_ = 5 *μ*M). Because carteramine A has no structural resemblance to known compounds that inhibit neutrophil chemotaxis, their finding provides a “novel platform to develop a new class of anti-inflammatory agents” [26]. Some of the anti-inflammatory compounds from marine invertebrates are listed in Table 1.

## 4. Chronic Diseases

A chronic condition is a human health condition or disease that is persistent or otherwise long lasting in its effects. The term chronic is usually applied when the course of the disease lasts for more than three months. Data from the World Health Organization show that chronic disease is also the major cause of premature death around the world. Common chronic diseases include asthma, arthritis, cancer, diabetes, heart diseases, and AIDS. Although chronic diseases are among the most common and costly health problems, they are also preventable and most can be effectively controlled. Apart from available treatment and intervention of drugs from various sources, marine derived compounds play a major role in the health benefits. Therefore, here we discuss the role of marine invertebrate derived natural products for some of the chronic diseases.

### 4.1. Cardiovascular Disease

Cardiovascular disease (CVD) is a class of diseases that affect the heart, blood vessels (arteries and veins), and blood circulation and is one of the leading causes of mortality and morbidity worldwide. Examples of CVD include atherosclerosis, CHD, stroke, heart failure, deep vein thrombosis, and peripheral arterial disease. The amount of fat in the diet and the type of fatty acids consumed can influence the likelihood of CVD and its risk factors [18]. High blood pressure (hypertension) is one of the major independent risk factors for CVD [33]. Significant discoveries have resulted primarily from analyzing natural products. Marine organisms such as invertebrates derived natural products have pharmacological properties [34]. Eryloside F from sponge *Erylus formosus* was found to be a potent thrombin receptor antagonist [35]. Thrombin receptor activation is likely to play a key role not only in arterial thrombosis but also in atherosclerosis [36]. Atherosclerosis starts with damage to the endothelium and subsequent deposition of fats, cholesterol platelets, cellular waste products, calcium, and other substances in the artery wall. These may stimulate endothelial cells to produce a vascular cell adhesion molecule that results in further buildup of cells and shrinkage of the arterial diameter [37]. Halichlorine from sponge *Halichondria okadai* is an inhibitor for the expression of vascular cell adhesion molecule 1 [38] and may thus impede atherogenesis [39].

### 4.2. Diabetes

Diabetes mellitus is a most serious and chronic disease whose incidence rates are increasing with increasing levels of obesity and also with aging of the general population over the world. Currently, an estimated 150 million people worldwide have diabetes and that this will increase to 300 million by 2025 [40]. Globally, type II diabetes (non-insulin dependent diabetes mellitus) accounts for greater than 90% of the cases [41, 42]. Research for novel anti-diabetic drugs to complement those in present clinical use has intensified over the years [43]. Callyspongynic acid is a polyacetylenic acid isolated from sponge *Callyspongia truncata* inhibits *α*-glucosidase [44]. *α*-Glucosidase inhibitors interfere with the hydrolysis of glycogen, keeping the glucose concentration in the blood at a lower level, and can be used to treat patients with diabetes l [45]. A polybromodiphenyl ether compound isolated from an Indonesian marine sponge *Lamellodysidea herbacea* inhibits protein tyrosine phosphatase 1B, an important target for diabetes treatment [46].

### 4.3. Arthritis and Osteoporosis

Arthritis describes a condition involving inflammation of the joints and is a disease affecting mostly the aged population. Preventing inflammation with its associated pain and reduced mobility symptoms is a primary requirement in arthritis treatment [47]. The alkaloid hymenialdisine isolated from marine sponge *Stylissa massa* inhibits proteoglycan degradation in bovine articular cartilage [48]. Osteoporosis is a multifactorial progressive skeletal disorder characterized by reduced bone mass and deterioration of bone microarchitecture, predisposing to increased fracture risk [49]. Osteoporosis is called a “silent disease” because it progresses without symptoms until a fracture occurs. Because of larger skeletons and no period of rapid hormonal change, osteoporosis progresses more slowly in men than women [50]. In the recent days, much attention has been paid for marine compounds for osteoporosis treatment [51]. Norzoanthamine is one of the zoanthamine classes of marine alkaloids isolated from a colonial zoanthid (cnidarians), *Zoanthus sp*. [52]. Norzoanthamine could protect skeletal proteins, such as collagen and elastin in the host animal bodies from external stresses and possibly enhance survival as it may be a promising drug candidate for osteoporosis treatment and prevention [53].

### 4.4. Neurodegenerative Diseases

Neurodegeneration is the term for the progressive loss of structure or function of neurons, including death of neurons. Many neurodegenerative diseases including Alzheimer's disease, Parkinson's disease, and Huntington's disease occur as a result of neurodegenerative processes. Current interventions for Alzheimer's disease (AD) include acetylcholinesterase inhibitors (AchI), which are indicated for patients with mild to moderate symptoms. A spectrum of alternative treatments for AD has also been proposed and must be examined judiciously in preclinical, clinical, and evidence-based research (EBR) studies [54]. Therefore, search for acetylcholinesterase inhibitors is useful for the treatment of Alzheimer's disease. Pharmacological studies with marine compounds affecting the nervous system involved three areas of neuropharmacology: the stimulation of neurogenesis, the targeting of receptors, and other miscellaneous activities on the nervous system. A new stigmastane type steroidal alkaloid 4-acetoxy-plakinamine B isolated from a Thai marine sponge *Corticium sp*. significantly inhibited acetylcholinesterase (IC_50_ = 3.75 *μ*M). This compound is reported to be the “first marine derived acetyl cholinesterase-inhibiting steroidal alkaloid” [55]. The inflammatory component to the pathology of neurodegeneration was most notably in Alzheimer's disease but also in Parkinson's disease and motor neuron disease [56]. Hymenialdisine is an alkaloid isolated from marine sponges, such as *Acanthella aurantianca* and *Stylissa massa* [57]. Hymenialdisine inhibits phosphorylation of the protein tau (which is hyperphosphorylated in Alzheimer's disease) with promising potential against human neurodegenerative diseases [58]. 11-Dehydrosinulariolide was obtained from formosan soft coral, *S. flexibilis, *promoting neuroprotective properties as a promising candidate for the treatment of Parkinson's disease [59].

### 4.5. HIV/AIDS

In order to combat the human immunodeficiency virus (HIV), diverse strategies have been developed to research on compounds which can be developed as therapeutic agents. Complementary and alternative medicine (CAM) can be defined as any treatment used in conjunction (complementary) or in place of (alternative) standard medical treatment [60]. More research is also required on the harmful and beneficial effects of concurrent CAM and treatment for HIV/AIDS. However, for all other types of CAM, natural products may be the alternative medicinal use for HIV/AIDS. Among them, marine derived natural products may be useful for the treatment of HIV. The peptides tachyplesin and polyphemusin, which are highly abundant in hemocyte debris of the horseshoe crabs *Tachypleus tridentatus*, *Limulus polyphemus* and the sponge metabolites avarol, avarone, ilimaquinone and several phloroglucinols has anti HIV activity [61, 62]. Avarol inhibits HIV by completely blocking the synthesis of the natural UAG suppressor glutamine transfer tRNA. Avarol inhibits HIV by almost completely blocking the synthesis of the natural UAG suppressor glutamine transfer tRNA. Synthesis of this tRNA is upregulated after viral infection and important for the synthesis of a viral protease, which is necessary for viral proliferation [63].

Clathsterol, a novel and active sulfated sterol from the Red Sea sponge *Clathria* sp., has been shown that inhibits HIV-1 RT at 10 *μ*M concentration [64]. An HIV-inhibitory cyclic depsipeptide microspinosamide, isolated from the marine sponge *Sidonops microspinosa,* inhibits HIV-1 infection in cell based *in vitro* assays [65]. A new polycyclic guanidine alkaloid, crambescidin 826, was reported from the marine sponge *Monanchora sp*. and it inhibits HIV-1 envelope-mediated fusion *in vitro *(IC_50_ = 1–3 *μ*M); it suggested that this compound might be the design of small molecule HIV-1 fusion inhibitors [66]. A new C22 furanoterpene (dehydrofurodendin) was isolated from the *Madagascan Lendenfeldia* sponge, active against HIV-1 RT-associated RNA- and DNA-directed DNA polymerase (IC_50_ = 3.2–5.6 *μ*M) [67]. Neamphamide A was isolated from the Papua New Guinea marine sponge *Neamphius huxleyi* that inhibits the cytopathic effect of HIV-1 infection in cell based *in vitro* assays (EC_50_ = 28 nM) [68]. Two bisquinolizidine alkaloids, petrosin and petrosin A was isolated from the Indian marine sponge *Petrosia similes* inhibits HIV-1 replication, formation of giant cells and recombinant reverse transcriptase *in vitro* [69]. Didemnaketals A and B were isolated from the ascidian *Didemnum sp*. and found to be inhibitors of HIV-1 protease [70]. Bioassay-guided fractionation of extracts of the Palauan ascidian *Didemnum guttatum* led to the isolation of cyclodidemniserinol trisulfate as an inhibitor of HIV-1 integrase [71]. Lamellarins was first isolated from prosobranch mollusks of the genus Lamellaria and subsequently obtained from *Didemnid ascidians* [72]. Lamellarin inhibits the integrase terminal cleavage activity and strands transfer activity [73]. The peptides Tachyplesins I–III and polyphemusins I and II, which are highly abundant in hemocyte debris of the horseshoe crabs *Tachypleus tridentatus* and *Limulus polyphemus*, respectively, were found to be HIV cell fusion inhibitors [74, 75]. Some of the marine natural products for chronic diseases are listed in Table 2.

### 4.6. Cancer

Many potent natural products which display effective anticancer activities have been discovered in the marine environment. Indeed, since the early 1990s, there has been a dramatic increase in the number of preclinical anticancer compounds from marine sources that have entered human clinical trials [14, 76]. Marine derived natural products from marine invertebrates are the valuable sources for anticancer drugs. Trabectidin (Yondelis), originally isolated from the Caribbean marine tunicate *Ecteinascidia turbinate*, has been approved for use as an anticancer agent in Europe [77, 78]. The compound was selected for clinical development on the basis of its novel chemical structure and its striking activity against tumor cell lines of different origins *in vitro* and *in vivo* models. In 2007, trabectedin obtained marketing authorization from the European Commission for the treatment of patients with advanced soft tissue sarcoma. In 2009, it received marketing authorization from the European Commission in combination with pegylated liposomal doxorubicin for the treatment of patients with relapsed platinum sensitive ovarian cancer. Clinical activity is currently being evaluated in other neoplasms, including prostate and breast cancer. Trabectedin's mechanism of action seems to be different from that of the available DNA damaging agents used in cancer chemotherapy to date. The cytoskeleton is also an interesting target for cancer therapy, as the microtubules and microfilaments are involved in cellular organization during cell division. A number of compounds from marine sponges and ascidians are microtubule inhibition [79]. Kahalalide F, a cyclic depsipeptide from the herbivorous marine mollusk, *Elysia rufescens*, has shown to be effective against cancer cell lines with strong multidrug resistance and against cell lines resistant to topoisomerase II inhibitors. *In vivo* models have also confirmed anticancer activity in various solid tumor models [80, 81].

The heteronemin, a marine sesterterpene isolated from the sponge *Hyrtios sp.,* inhibits NF-*κ*B activation and activates both initiator caspase-8 and caspase-9, which are implicated in the extrinsic and intrinsic apoptotic pathway, respectively, in chronic myelogenous leukemia cells [82]. Tyrindoleninone and 6-bromoisatin are indole derivatives from marine mollusk *Dicathais orbita* that induces apoptosis in female reproductive cancer cell lines ovary, granulosa, and choriocarcinoma (OVCAR-3, KGN, Jar), respectively [83]. Makaluvamine A is a pyrroloquinoline, principally isolated from the sponge *Zyzzya fuliginosa* that has potent anticancer activity in HCT-116 cells [84]. Ascididemin (ASC), a aromatic alkaloid isolated from the Mediterranean ascidian *Cystodytes dellechiajei* [85], which are strong inducer of apoptosis in HL-60 and P388 leukemia cells [86]. An alkaloid Lamellarin D (LAM-D), initially isolated from a prosobranch mollusc of the genus *Lamellaria*, exhibits cytotoxicity against many different tumors. LAM-D potently stabilizes topoisomerase I DNA covalent complexes to be promoting the formation of DNA single strand breaks. LAM-D also promotes nuclear apoptosis in leukemia cells via the intrinsic apoptotic pathway [87, 88]. Spongistatin 1 a macrocyclic lactone isolated from the marine sponges *Spirastrella spinispirulifera* and *Hyrtios erecta *induces apoptosis by interact with caspase dependent pathway by the release of cytochrome c, Smac/DIABLO, and Omi/HtrA2 from the mitochondria to the cytosol, leading to apoptosis in Jurkat cells [89]. Chitosan is produced commercially by deacetylation of chitin, which has the structural element in the exoskeleton of crustaceans (such as crabs and shrimp) and cell walls of fungi. Diethylaminoethyl chitosan induces apoptosis in HeLa cells via activation of caspase-3 and p53 expression [90]. Bryostatins are a group of macrolide lactones first isolated in the 1960s by George Pettit from extracts of a species of bryozoan, *Bugula neritina*. The structure of bryostatin 1 was determined in 1982. To date 20 different bryostatins has been isolated, those are currently under investigation as anti-cancer agents [91, 92]. Bryostatin 1 induces apoptosis in HL-60 chronic lymphocytic leukaemia and also acts synergistically in combination with other anti-cancer drugs.

Eribulin mesylate (E7389) is a microtubule dynamics inhibitor that is a simplified, synthetic analog of the marine natural macrolide halichondrin B, which was first isolated from the Japanese sponge *Halichondria okadai *[93] and subsequently from several unrelated sponges belonging to the *Axinella* family. Eribulin mesylate is now in phase I clinical studies exploring weekly and 3-week schedule [94]. Aplidine is a cyclic depsipeptide extracted from the ascidian *Aplidium albicans* [95]. It is a member of the class of compounds known as didemnins. Aplidin is a novel cyclic depsipeptide, currently in phase II/III clinical trials for solid and hematologic malignancies [96]. Hemiasterlin is originally identified as natural products from marine sponges (*Cymbastela sp., Hemiasterella minor, Siphonochalina sp., and Auletta sp*.), comprising a small family of naturally occurring tripeptides containing three highly modified amino acids [97, 98]. Hemiasterlin is a potent inhibitor of cell growth, depolymerizes microtubules, and arrests cells in the G2-M phase of the cell cycle [99]. Elisidepsin (PM02734, Irvalec) is a synthetic marine derived cyclic peptide of the Kahalalide F family currently in phase I and II clinical development [100, 101]. Among the various chemical class such as alkaloids, terpenoids, polyketide, macrocyclic lactone, peptide and proteins from marine environment including marine invertebrates could inhibits cell growth signal, induces apoptosis, inhibits invasion and metastasis of cancer [102–104]. The increasing interest in CAM among cancer patients may be due to limitations of conventional cancer treatment; therefore, marine invertebrate derived natural products are alternate way for the therapy for cancer. List of anticancer natural products is listed in Table 3.

## 5. Conclusion

There exists a great need for new, effective, and safe treatments of chronic diseases such as cancer, arthritis, osteoporosis, cardiovascular diseases, chronic inflammation, and Alzheimer's disease. Marine invertebrate derived natural products provide an excellent opportunity to study diverse and unique compounds not readily accessible from any other source leading to expansion of the pharmaceutical pipeline. Novel products from marine invertebrates exhibit potent activity in various *in vitro* and *in vivo* assays geared towards discovering pharmaceutical leads in these areas. The identification and development of natural compounds and their derivatives have greatly contributed to this progress and many of these compounds are now being used in clinical practice. Nature is still today a rich source of active principles against various diseases. Research results both testify to the evolution of knowledge coming from pharmacognosy and its historical roots in ancient medicine, and to the great possibilities of future progress by means of a rational, natural product-based drug discovery approach. Marine invertebrate's derived natural products will become a more significant part of the pipeline and alternate medicines for anti-inflammatory and chronic diseases.

## Figures and Tables

**Table 1:** 

Name of the compound	Chemistry	Name (species)	Target effects	References
Ascidiathiazone	Alkaloids	Ascidian (*Ascidian Aplidium*)	Anti-inflammatory action in human neutrophils	[27]
Cembranolides	Cembranoids	Soft coral (*Lobophytum crassum*)	Inhibitors of COX-2	[29]
Durumolides	Cembranoids	Soft coral (*Lobophytum duru*)	Inhibitors of iNOS and COX-2	[30]
Frajunolides	Diterpenoids	Gorgonian (*Junceella fragilis*)	Anti-inflammatory action in human neutrophils	[31]
Manzamine	Alkaloids	*Sponge *sp.	Inhibitors of thromboxane B2	[25]
Plakortide P	Polyketide	Sponge (*P. angulospiculatus*)	Antineuroinflammatory	[32]
Rubrolide O	Halogenated furanone	Ascidian (*Synoicum *sp.)	Anti-inflammatory action in human neutrophils	[28]
Carteramine A	Alkaloid	Sponge (*Stylissa carteri*)	Inhibit neutrophil chemotaxis	[26]

**Table 2:** List of some marine invertebrate derived natural products for chronic diseases.

Name of the compound	Chemistry	Name (species)	Target effects	References
Eryloside F	Penasterol disaccharide	Sponge (*Erylus formosus*)	Atherosclerosis	[35]
Halichlorine	Alkaloid	Sponge (*Halichondria okadai*)	Atherosclerosis	[38]
Callyspongynic acid	Polyacetylenic acid	Sponge (*Callyspongia truncate*)	Diabetes	[44]
Hymenialdisine	Alkaloid	Sponge (*Stylissa massa*)	Arthritis	[48]
Norzoanthamine	Alkaloid	Cnidarians, *Zoanthus *sp.	Osteoporosis	[53]
4-Acetoxy-plakinamine B	Alkaloid	Sponge (*Corticium *sp.)	Acetylcholinesterase inhibition	[55]
Hymenialdisine	Alkaloid	Sponge *Acanthella aurantiaca* and *Stylissa massa *	Alzheimer's disease	[57]
11-dehydrosinulariolide	Cembranolide	Soft coral (*S. flexibilis*)	Parkinson's disease	[59]
Avarol	Sesquiterpene hydroquinone	*Sponge *sp.	HIV	[63]
Clathsterol	Depsipeptide	Sponge (*Clathria *sp.)	HIV	[65]
Crambescidin 826	Alkaloid	Sponge (*Monanchora *sp.)	HIV	[66]
Dehydrofurodendin	Furanoterpene	Sponge (*Madagascan Lendenfeldia*)	HIV	[67]
Neamphamide A	Depsipeptide	Sponge (*Neamphius huxleyi*)	HIV	[68]
Lamellarins	Alkaloid	Mollusks (*Lamellaria *sp.)	HIV	[73]
Tachyplesins	Peptide	Horseshoe crabs *Tachypleus tridentatus* and *Limulus polyphemus *	HIV	[75]

**Table 3:** List of some anticancer natural products from marine invertebrates.

Name of the compound	Chemical class	Source of organisms	Targets	Reference
Trabectidin	Alkaloids	Tunicate (*Ecteinascidia turbinate*)	Anticancer effect in breast and prostate and so forth.	[77, 78]
Kahalalide F	Cyclic depsipeptide	Mollusk (*Elysia rufescens*)	Inhibits topoisomerase II in cancer	[81]
Heteronemin	Sesterterpene	Sponge (*Hyrtios *sp.)	Inhibits Leukemia (K562 cells) cancer cells	[82]
Tyrindoleninone and 6-bromoisatin	Indole derivative	Mollusk (*Dicathais orbita*)	Inhibits ovary, granulosa, choriocarcinoma (OVCAR-3, KGN, Jar) cells	[83]
Makaluvamine A	Pyrroloquinoline	Sponge (*Zyzzya fuliginosa*)	Colon cancer (HCT-116 cells) inhibition	[84]
Ascididemin	Alkaloid	Mediterranean ascidian (*Cystodytes dellechiajei*)	Inhibits leukemia (HL-60 and P388) cancer cells	[85, 86]
Lamellarin D	Alkaloid	Prosobranch mollusc of the genus (*Lamellaria*)	Inhibits leukemia cancer cells	[87, 88]
Spongistatin 1	Macrocyclic lactone	Sponges (*Spirastrella spinispirulifera *and* Hyrtios erecta*)	Inhibits leukemia (Jurkat) cancer cells	[89]
Chitosan	Polysaccharides	Crabs and shrimp (various species)	Inhibits and induces apoptosis of HeLa cancer cell	[90]
Bryostatins	Macrolides	Bryozoan (*Bugula neritina*)	Inhibits various cancer types and currently used in clinical trials	[91]
Eribulin mesylate (E7389)	Macrolide	*Halichondria okadai* and *Axinella* family	Microtubule inhibitor and presently used in clinical trials	[93]
Aplidin	Cyclic depsipeptide	Ascidian (*Aplidium albicans*)	Currently in phase II/III clinical trials for solid and hematologic malignancies	[96]
